# Photoaging and actinic keratosis in Danish outdoor and indoor workers

**DOI:** 10.1111/phpp.12451

**Published:** 2019-02-20

**Authors:** Kasper Grandahl, Jonas Olsen, Kersti Brosbøl Engelund Friis, Ole Steen Mortensen, Kristina Sophie Ibler

**Affiliations:** ^1^ The Department of Occupational Medicine Copenhagen University Holbaek Holbaek Denmark; ^2^ The Department of Dermatology Zealand University Hospital Roskilde Roskilde Denmark; ^3^ Department of Public Health Section of Social Medicine University of Copenhagen Copenhagen Denmark

**Keywords:** actinic keratosis, facial wrinkles, outdoor work, photoaging, ultraviolet radiation exposure

## Abstract

**Background:**

The risk of photoaging and actinic keratosis caused by work related solar ultraviolet radiation exposure has not previously been investigated in Nordic countries. The objectives of this study were to describe the occurrence of photoaging, actinic keratosis, and keratinocyte cancer in a population of Danish outdoor and indoor workers, and investigate the association between these clinical findings and semi‐objective measures of work related solar ultraviolet radiation exposure in the same population.

**Methods:**

A clinical cross‐sectional study of the occurrence of facial wrinkles, actinic keratosis, keratinocyte cancer, and melanocytic nevi in a population of Danish outdoor and indoor workers and associations with semi‐objective measures of work related solar ultraviolet radiation exposure based on a combination of dosimetry and self‐report.

**Results:**

Work related solar ultraviolet radiation exposure was significantly positively associated with occurrence of facial wrinkles (α = 0.05). Actinic keratosis was associated to status as outdoor worker (OR = 4.272, CI [1.045‐17.471]) and age (*P* < 0.001, CI [1.077‐1.262]) and twice as common in outdoor workers (10.3% CI [0.05, 0.15]) compared to indoor workers (5.1% CI [0.00, 0.10]). Only two cases of keratinocyte cancer were diagnosed (<1%). Older age was negatively associated with occurrence of melanocytic nevi.

**Conclusion:**

Outdoor work in Denmark is associated with increased occurrence of facial wrinkles and actinic keratosis from solar ultraviolet radiation exposure, thus justifying sun safety at Danish workplaces from a clinical perspective.

## 1 INTRODUCTION

Skin aging results from both the passage of time (intrinsic aging) and from cumulative exposure to external factors (extrinsic aging).^1^ Photoaging is defined as premature aging of the skin caused by exposure to ultraviolet radiation (UVR).^2^, ^3^ UV‐A penetrates to the dermis causing indirect damage to collagen and elastin by a photochemical process, and UV‐B penetrates to the epidermis and upper dermis causing direct DNA and RNA damage.^4^, ^5^ Clinical signs of photoaging include loss of elasticity, dyspigmentation, skin thinning, and facial wrinkles (FW).^6^ Excessive exposure to UV‐B radiation is known to induce multiple cascades of molecular signaling events in skin cells that produce inflammation, immunosuppression, failure of apoptosis, and aberrant differentiation. Cumulatively, these events result in mutagenesis and, ultimately, carcinogenesis.^7^ Thus, UVR exposure increases the risk of developing actinic keratosis (AK) and melanocytic nevi (MN)^8^, ^9^, ^10^ and is the main etiological cause of keratinocyte cancer (KC).^4^, ^11^, ^12^ Other known risk factors include older age and fair skin type for AK and FW,^13^, ^14^, ^15^ male sex and exposure to tar products, welding, poly‐aromatic hydrocarbons (PAH), arsenic for AK^15^, ^16^, ^17^ and smoking for FW.^18^, ^19^ Conversely, age is negatively associated with MN count.^20^ Some papers report that clinical actinic elastosis and wrinkles may have a protective effect against development of basal cell carcinoma; however, the collagen repair processes in chronic and/or intermittent UVR exposure are yet not fully understood.^21^, ^22^

Work related solar UVR exposure has been found to be associated with increased prevalence of AK in European seafarers above the age of 40 years 23 and increased severity of FW in Italian farmers and Polish outdoor workers.^24^, ^25^ In Denmark, it was recently shown that people working predominantly outdoor were substantially more exposed to solar UVR compared to people working indoor^26^ and thus at a higher risk of developing photoaging, AK, and KC. No previous studies have associated objective measures of work related solar UVR exposure based on dosimetry with clinical signs of photoaging, AK, and KC in a Northern country like Denmark.

### 1.1 Objective

1. To investigate the occurrence of FW, AK, KC, and MN in a population of Danish outdoor and indoor workers.
2. To investigate the association between occurrence of FW, AK, KC, and MN and semi‐objective measures of work related solar UVR exposure in a population of Danish outdoor and indoor workers.

## 2 METHODS

### 2.1 Study design

This is a cross‐sectional study investigating the occurrence of FW, AK, KC, and MN and the association with work related solar ultraviolet radiation exposure in a population of Danish outdoor and indoor workers.

### 2.2 Recruitment

The participants, 234 in all, were recruited from a cohort of Danish outdoor and indoor workers participating in the project “Occupational skin cancer” in 2016.^26^, ^27^, ^28^ From the cohort, 322 workers were offered participation aiming at a 1:2 indoor/outdoor worker ratio. Hereof 66 declined, canceled, or did not show up for the skin examination, and another 22 failed to complete the questionnaire, resulting in a participation rate of 72.7% (234 of 322). Outdoor professions included construction‐, postal‐, road‐ and dockworkers, gardeners, roofers, masons, and unskilled laborers. Indoor workers included porters, administration workers, surveyors, crane technicians, and blacksmiths. Among carpenters, half reported their profession as outdoor and the other half as indoor.

### 2.3 Survey data

Self‐reported data were obtained from a questionnaire study and included information about age; sex; smoking; skin type according to Fitzpatrick scale^29^; regular work related exposure or not to tar products, welding, PAHs, and arsenic; work status as outdoor or indoor worker; and number of years in current professions and previous jobs.^28^

### 2.4 Skin examination

All 234 participants underwent a standardized clinical skin examination by two trained investigators, recording occurrence of FW, AK, KC, and MN between August and December 2016. The investigators were blinded to the participants’ occupation. Dermoscopy was used in lesions suspicious of AK or KC, and lesional punch biopsies were performed if KC was suspected. Diagnosis of KC was based on histology. FW were rated on a validated scale from one (least severe) to five (most severe) in the periorbital region reflecting static wrinkles (FW static periorbital) and dynamic wrinkles (FW dynamic periorbital) as well as in the neck reflecting static wrinkles (FW static neck).^30^ MN (Ø > 2 mm) were counted on the left forearm as a predictor of the number of MN on the whole body.^31^ As part of a different study on the blood vessel morphology of the skin, the participants had optical coherence tomography (OCT) scans performed. These results are reported elsewhere.^32^

### 2.5 Work related solar UVR exposure

General measures of semi‐annual (between April and September) work related standard erythema dose (SED) for outdoor workers and each of seventeen different professions 26, 27 were combined with self‐reported number of work years in current profession and in previous jobs as outdoor worker to assess the total work related SED of each participant:

$$\begin{array}{cc} \text{Total}\;{\text{SED}}_{\mathrm{work}\mathrm{related}} & ={\text{SED}}_{\mathrm{curr}\mathrm{prof}}*\text{Work}\;{\text{years}}_{\mathrm{curr}\mathrm{prof}}\\ & +{\text{SED}}_{\mathrm{out}\mathrm{work}}*\text{work}\;{\text{years}}_{\mathrm{prev}\mathrm{out}\mathrm{work}} \end{array}$$

Total SED_work related_: total work related solar UVR exposure; SED_curr_ _prof_: semi‐annual SED, current profession; Work years_curr_ _prof_: number of work years, current profession; SED_out work_: semi‐annual SED, outdoor workers; Work years_prev out work_: number of years, previous jobs as outdoor worker.

### 2.6 Statistical analysis

Mean values were used for continuous variables ± SD if normally distributed, and median values were used for non‐normally distributed and ordinal variables (IQR). Binary variables were reported as percentage (count). Comparisons between groups were made using chi‐square (χ^2^) test for nominal and ordinal variables, Mann‐Whitney *U* test for non‐normally distributed continuous variables and independent samples *t* test for normally distributed continuous variables. Spearman bivariate correlation and partial correlation were used to test associations, assuming ordinal distribution of data. Since our study was an unmatched case‐control study, an unconditional logistic regression model was used to predict the odds ratio for AK risk factors related to UVR exposure.^33^ In the logistic regression analysis, Total SED_work related_ was scaled in units of 100 SED in order to get a meaningful odds ratio statistic per unit change. Statistical significance was determined using α = 0.05. IBM SPSS version 24 (SPSS Inc., Chicago, IL, USA) was used for data analysis.

### 2.7 Ethics

The Region Zealand Ethical Scientific Committee and Data Monitoring Authority approved the study. File numbers: SJ‐509 and REG‐130‐2015. The study was conducted in accordance with the principles of the Declaration of Helsinki.

Informed consent was obtained from all participants.

## 3 RESULTS

Data from all 234 participants were available for analysis. There were 155 outdoor workers and 79 indoor workers.

The participants were mostly males (72.6%) with a mean age of 48 years (range 17‐71) and skin types II‐IV (94.8%). Outdoor professions included gardeners (31.8%), unskilled laborers (14.3%), road workers (7.8%), dockworkers (6.5%), carpenters (5.2%), and roofers (3.9%). Indoor professions included porters (34.8%), administrative workers (16.5%), crane technicians and/or blacksmiths (8.9%) and carpenters (8.9%).

Missing data from the skin examination included 7 cases of FW rating in the neck (six outdoor workers and one indoor worker) and 26 cases of MN count (nineteen outdoor and seven indoor workers). This was due to registration error and late introduction of MN count as part of the skin examination.

Table 1 provides an overview of the total population and groups of outdoor and indoor workers with respect to background characteristics and risk factors.

**Table 1:** Background characteristics and risk factors in the total population and groups of outdoor and indoor workers

	Total population (234)	Outdoor workers (155)	Indoor workers (79)
Age (years, ±SD)	47.6 ± 11	47.1 ± 11	48.6 ± 10
Sex (% of males, n)	72.6% (170)	69.0% (107)	79.9% (63)
Smoking (% never, n)	54.7% (128)	53.5% (83)	57.0% (45)
Skin type (%, n)			
I	2.6% (6)	1.9% (3)	3.8% (3)
II	26.9% (63)	28.4% (44)	24.1% (19)
III	44.4% (104)	43.9% (68)	45.6% (36)
IV	23.5% (55)	23.9% (37)	22.8% (18)
V	2.6% (6)	1.9% (3)	3.8% (3)
Tar products (%, n)	9.0% (21)	10.3% (16)	6.3% (5)
Welding (%, n)^a^	10.3% (24)	6.5% (10)	17.7% (14)
Total SED (mean, ±SD)^a^	3613 ± 2596	4147 ± 2628	2435 ± 1997

a Significantly different between groups of outdoor and indoor workers (α = 0.05).

### 3.1 Exposures

Work related solar UVR exposure was significantly higher in outdoor worker (*P* < 0.001). Exposure to PAH was only reported by one participant, whereas arsenic was not reported by any.

Exposure to welding was significantly more common in indoor workers (explained by the relatively high proportion of crane technicians and/or blacksmiths in this group) compared to outdoor workers (*P* = 0.007).

### 3.2 Keratinocyte cancer

Only two participants (<1%) were diagnosed with KC (basal cell carcinomas). One was a 53‐year‐old male outdoor worker with skin type II and a Total SED_work related_ almost twice that of the mean for the total population. The other was a 54‐year‐old female indoor worker with skin type I and a Total SED_work related_ that was near negligible.

### 3.3 Actinic keratosis

Twenty of 234 cases were diagnosed with AK, corresponding to 10.3% CI [0.05, 0.15] outdoor workers (16 cases) and 5.1% CI [0.00, 0.10] indoor workers (4 cases). Most cases of AK were diagnosed in workers above the age of 48 years (18 cases); in this age group, the proportion of AK by sex was in the total population: males 17.2% CI [0.09, 0.25], females 6.5% CI [0.00, 0.16], outdoor workers: males 21.4% CI [0.10, 0.33] females 8.7% CI [0.00, 0.21], and indoor workers: males 10.8% CI [0.00, 0.21] (no females). Chi‐square (χ ^2^) revealed no significant differences in occurrence of AK between outdoor and indoor workers.

**Figure 1:**
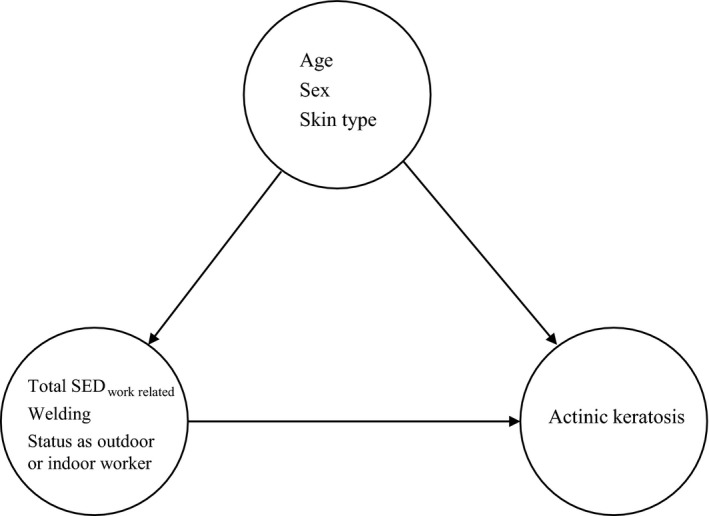
Actinic keratosis risk factors in the logistic regression model illustrated as exposure or confounder

Known risk factors from other studies predicting AK development (stated in the introduction and illustrated in Figure 1) were entered into the logistic regression model collectively: age, Total SED_work related_ (scaled in units of 100 SED), sex, exposure to welding, skin type, and status as outdoor or indoor worker. The model showed a statistically significant increase in OR of 1.166 per year of age (*P* < 0.001, CI [1.077‐1.262]) as well as a statistically significant higher OR of 4.272 (*P* = 0.043, CI [1.045‐17.471])—equivalent to an estimated RR of 3.977—in outdoor workers.^34^ The remaining predictors Total SED_work related_, sex, exposure to welding and skin type were insignificant in the model. The crude and adjusted OR of all predictors in the logistics regression model is presented in Table 2. Further analysis, separating the exposures “Total SED_work related,_” “exposure to welding,” and “status as outdoor or indoor worker” each in logistic regression models with age, sex, and skin type as confounders did not change significance of results.

**Table 2:** OR (95% CI) crude and adjusted for all predictors for AK in the logistics regression model

Predictors for AK	Crude OR (95% CI)	Adjusted OR (95% CI)
Age	1.140 (1.067‐1.217)	1.166 (1.077‐1.262)
Total SED_work related_ a	1.015 (0.999‐1.032)	0.991 (0.971‐1.012)
Male sex	3.671 (0.827‐16.296)	3.916 (0.806‐19.018)
Exposure to welding	0.437 (0.056‐3.419)	0.664 (0.072‐6.091)
Skin type		
I	1	1
II	0.250 (0.039‐1.623)	0.514 (0.049‐5.380)
III	0.122 (0.019‐0.808)	0.164 (0.016‐1.677)
IV	0.200 (0.029‐1.378)	0.317 (0.028‐3.617)
V	<0.001 (0.000)	<0.001 (0.000)
Status as outdoor worker	2.158 (0.696‐6.688)	4.272 (1.045‐17.471)

a Total SED work related scaled in units of 100 SED.

### 3.4 Facial wrinkles

**Figure 2:**
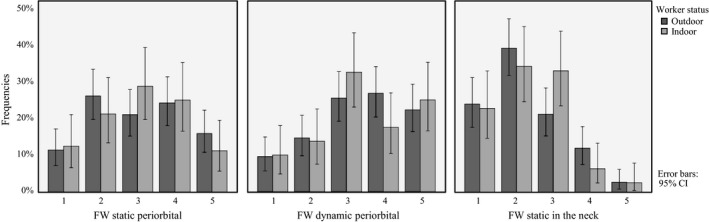
Frequencies including 95% CI for facial wrinkle (FW) ratings (1‐5) in outdoor and indoor workers

Figure 2 shows the frequencies including a 95% CI for FW ratings (1‐5) in outdoor and indoor workers in three separate bar charts, one for each of FW _static periorbital_, FW_dynamic periorbital_, and FW_static in the neck_.

Table 3 shows the differences in Total SED_work related_ between each group of FW ratings. We found a significant positive association between FW and Total SED_work related_ using a Spearman's partial correlation adjusting for age, smoking, and skin type, which is also shown in Table 2.

**Table 3:** FW ratings (1‐5) with number (n), Total SED work related (mean ±SD) and the results of partial correlation

	Rating	n	Total SED_work related_ ±SD	*r*	*p*
FW_static periorbital_	1	28	1522 (±1182)		
	2	58	3012 (±2071)		
	3	56	3416 (±2340)	0.185	0.005
	4	58	4668 (±2774)		
	5	34	4880 (±2931)		
FW_dynamic periorbital_	1	23	1393 (±1101)		
	2	34	2892 (±2101)		
	3	66	2967 (±2133)	0.195	0.003
	4	56	4726 (±2470)		
	5	55	4628 (±2988)		
FW_static neck_	1	54	2728 (±2426)		
	2	86	3367 (±2348)		
	3	58	3639 (±2547)	0.121	0.070
	4	23	5498 (±2817)		
	5	6	6085 (±2944)		

Adjusting for age, smoking, and skin type presented as correlation coefficient (*r*) and *P*‐value (*P*).

### 3.5 Melanocytic nevi

The median (IQR) MN count was 4 (6) in the study population. For outdoor workers, it was 4 (6) and indoor workers 3 (8). We found no significant difference in MN count using Mann‐Whitney *U* test comparing outdoor and indoor workers.

Results of Spearman's partial correlation showed a significantly negative association between MN count and age adjusting for Total SED_work related_ (*r* = **−**0.189, *P* = 0.006).

## 4 DISCUSSION

In Nordic countries, high levels of solar UVR exposure at work 26 are likely to contribute to photoaging and AK. Our finding of a positive association between FW and work related solar UVR exposure, when adjusting for age, smoking habits, and skin type, certainly implies that work related solar UVR exposure is an independent risk factor for the development of photoaging in Danish outdoor workers. In addition, Danish outdoor workers had an increased risk of developing AK compared with indoor workers. Similarly, AK in outdoor workers (10.3% CI [0.05, 0.15]) was twice as common compared with indoor workers (5.1% CI [0.00, 0.10]).

The prevalence of AK in male outdoor workers above the age of 48 years (21.4% CI [0.10, 0.33]) found in our study was lower than the previously reported prevalence in male European seafarers above the age of 50 years of 29.1%.^23^ This may be explained by differences in solar UVR exposure, mainly because of higher exposure levels in Central and Southern Europe compared to Northern Europe as annual levels of ambient solar UVR increases with decreasing geographic latitude 35 and partly because of increased reflection of solar UVR from the sea, and/or differences in sun safety behavior.^26^ In a population study from North‐west England, the prevalence of AK was 18.6% in males and 5.9% in females above the age of 50 years.^36^ In comparison, the higher prevalence of AK found in our study (21.4% CI [0.10, 0.33] in males, 8.7% CI [0.00, 0.21] in females, >48 years) is probably due to a relatively higher proportion of outdoor workers.

The negative association between MN count and age in our study is consistent with previous results in Australia 20 and the lack of association between MN count and work related solar UVR exposure is most likely due to a strong mediator effect of age.

The incidence of KC is generally well predicted by geographic latitude dependent levels of ambient solar UVR.^37^ Yet, the incidence of KC in Denmark is relatively high in global comparison.^38^, ^39^ In younger populations, KC and AK are both relatively rare and usually debut around or after retirement age.^40^, ^41^ Both occur with several years of latency, which may explain the somewhat low prevalence of KC and AK in our study population with a mean age of 48 years. It would be of great relevance to reexamine the cohort later in life and evaluate the future development of photoaging, AK, and KC and the association with the observed exposure to solar UVR.

### 4.1 Strengths and limitations

To our knowledge, this study is the first of its kind to describe the association between semi‐objective measures of work related solar UVR exposure based on a combination of dosimetry and self‐report and clinical signs of photoaging, AK, and KC in a population of outdoor and indoor workers of different professions.

Participants were sampled from a cohort of diverse outdoor and indoor professions broadly representing the intended target populations.^28^ However, selection bias from worker self‐selection or pre‐screening is possible and may affect the generalizability of results.

Participants in this study do not represent higher socioeconomic status professions, which has been associated with increased relative risk of KC of type cutaneous squamous cell carcinoma in previous cohort studies from the Nordic countries.^42^ Again, somewhat careful consideration should be given to generalizing the results.

The clinical examination of the participants was systematic, standardized, and performed by two investigators with dermatological expertise and thus minimizing the risk of inter‐observer variation.

The FW rating scale used in this study was originally developed and validated for rating crow's feet (periorbital).^30^ This may lead to poor validity as regards ratings of FW in the neck and explain the overall lower FW ratings in the neck compared to the periorbital region illustrated in Figure 2.

The semi‐objective measures of work related solar UVR exposure used in this study were based solely on UV‐B radiation and limited to the Danish summer season. However, UV‐A radiation contributes only very little to total work related solar UVR dose 43 and the intensity of solar UVR is quite low outside the Danish summer season.^44^ UV‐A radiation may still contribute to the development of photoaging and KC, as evidenced by its potential to induce elastin degeneration and DNA photolesions in the human basal epidermis suggested by experimental studies.^45^, ^46^, ^47^

Other sources of UVR exposure not accounted for in this study include outdoor stay at leisure and on sun holiday and use of sun beds. However, in the “Occupational skin cancer” cohort, the UVR exposure of outdoor and indoor workers was largely the same at leisure 26 as was the frequency of sun holidays and use of sun beds.^28^

The use of average SEDs in estimating individual Total SED_work related_ corresponds to the use of a job‐exposure‐matrix to assess exposure and carries the same risk of non‐differential exposure misclassification, due to between worker and within profession variation, with the risk of bias by under‐ or overestimating associations.^26^, ^48^, ^49^, ^50^ However, use of observed measures of exposure partially derived from the participants themselves minimizes the risk of information bias in this study.

The use of self‐reported number of work years in current profession and in previous jobs as outdoor worker to determine Total SED_work related_ may nevertheless constitute a source of information bias in this study, especially as almost 7% of participants reported a total number of work years in excess of their current age and had to be excluded. The categorization of outdoor and indoor workers based on self‐report may likewise be a source of information bias.

The entry of multiple predictors for AK in the logistic regression model at the same time implies a risk of bias from over‐adjustment, as some of these independent variables (predictors) not only correlate with the dependent variable (AK), but also to some degree with each other for example age and Total SED_work related_.^51^

## 5 CONCLUSION

Outdoor workers have more pronounced photoaging, as reflected by increased occurrence of FW and AK when compared to indoor workers. While this may not pose an occupational health hazard in itself, the well‐established association between photoaging and KC strongly implies that Danish outdoor workers are at long‐term risk of developing KC as a consequence of their job. Sun safety at Danish workplaces is therefore important. Likewise, it is important for healthcare professionals to report AK and KC in case of suspected work related disease, as required by Danish law, as it appears underreported.

## CONFLICT OF INTEREST

None.
